# Supplementary material for: The interactome of intact mitochondria by cross-linking mass spectrometry provides evidence for coexisting respiratory supercomplexes
Source: Mol Cell Proteomics. 2017 Dec 8;17(2):216–32. doi: 10.1074/mcp.RA117.000470 (PMC5795388; doi:10.1074/mcp.RA117.000470)
Supplement: Supplemental Data [file supp_RA117.000470_133922_0_supp_23978_fzffwf.zip › spectra_annotation/mito_spectra_annotation/246-1-4-1-9-1.pdf]

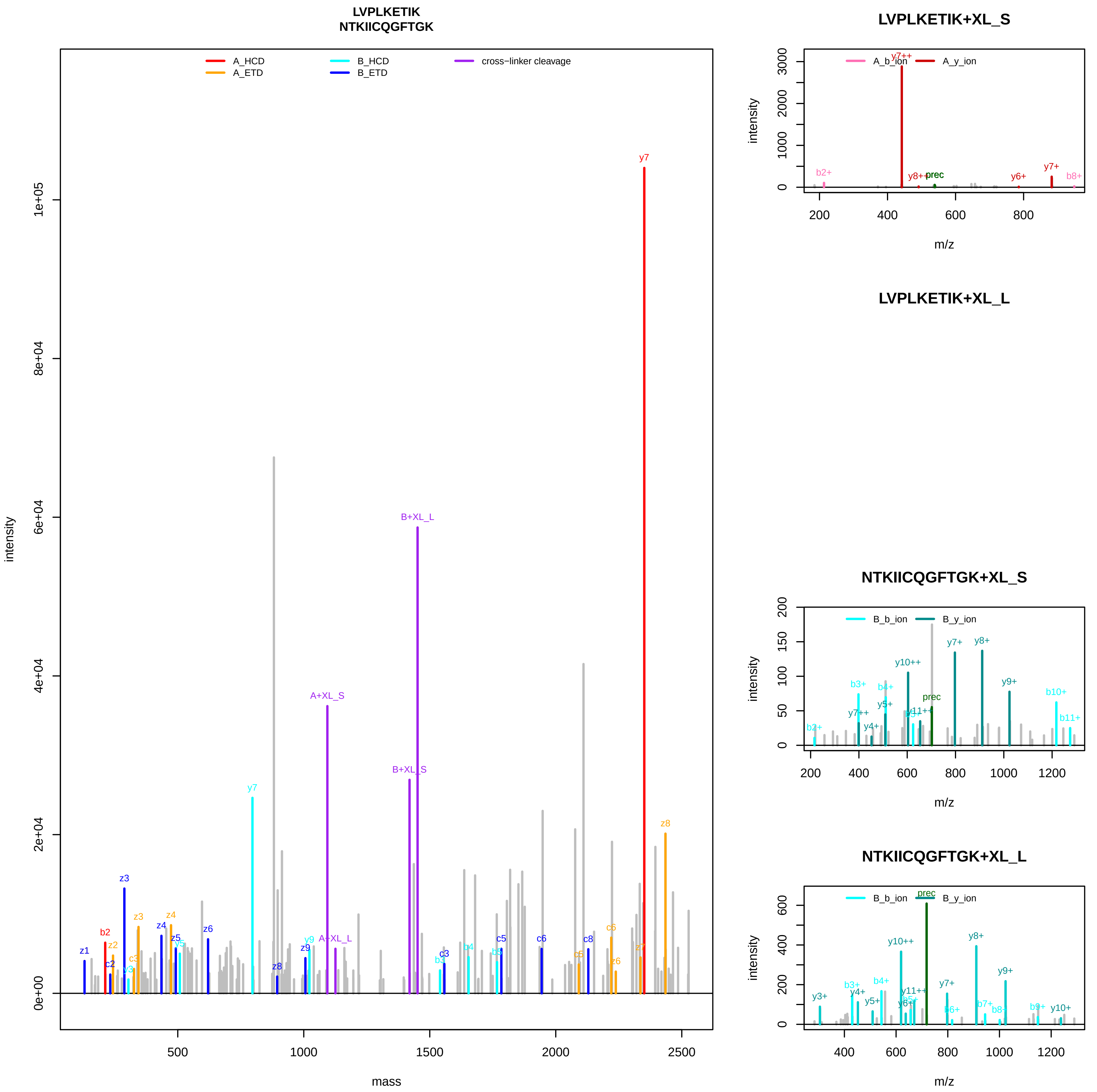
LVPLKETIK
NTKIICQGFTGK
A_HCD
A_ETD
B_HCD
B_ETD
cross−linker cleavage
intensity
mass
0e+00
2e+04
4e+04
6e+04
8e+04
1e+05
500
1000
1500
2000
2500
y7
B+XL_L
A+XL_S
B+XL_S
A+XL_L
z8
z3
z4
z6
z5
z2
z1
b2
c2
c3
y3
y5
y9
z9
b3
c3
b4
b5
c5
c6
c8
c5
c6
z6
z7
LVPLKETIK+XL_S
A_b_ion
A_y_ion
y7++
b2+
y8++
prec
y6+
y7+
b8+
m/z
LVPLKETIK+XL_L
NTKIICQGFTGK+XL_S
B_b_ion
B_y_ion
y10++
y7+
y8+
y9+
b3+
b4+
y5+
b10+
y7++
y4+
b11+
b2+
NTKIICQGFTGK+XL_L
y3+
b3+
y4+
y5+
b4+
y10++
y6+
y11++
y7+
b6+
b7+
y8+
b8+
y9+
b9+
y10+
